# *OoNAC72*, a NAC-Type *Oxytropis ochrocephala* Transcription Factor, Conferring Enhanced Drought and Salt Stress Tolerance in *Arabidopsis*

**DOI:** 10.3389/fpls.2019.00890

**Published:** 2019-07-11

**Authors:** Huirui Guan, Xin Liu, Fei Niu, Qianqian Zhao, Na Fan, Duo Cao, Dian Meng, Wei He, Bin Guo, Yahui Wei, Yanping Fu

**Affiliations:** Department of Life Science, Key Laboratory of Resource Biology and Biotechnology in Western China, Northwest University, Xi’an, China

**Keywords:** *O. ochrocephala*, NAC transcription factor, drought stress, salt stress, ABA hypersensitivity, transgenic *Arabidopsis*

## Abstract

The NAC proteins form one of the largest families of plant-specific transcription factors (TFs) and play essential roles in developmental processes and stress responses. In this study, we characterized a NAC domain transcription factor, *OoNAC72*, from a legume *Oxytropis ochrocephala*. OoNAC72 was proved to be localized in the nuclei in tobacco lower epidermal cells and had transcriptional activation activity in yeast, confirming its transcription activity. *OoNAC72* expression could be induced by drought, salinity and exogenous abscisic acid (ABA) in *O. ochrocephala* seedlings. Furthermore, over-expression of *OoNAC72* driven by CaMV35S promoter in *Arabidopsis* resulted in ABA hypersensitivity and enhanced tolerance to drought and salt stresses during seed germination and post-germinative growth periods. In addition, over-expression of *OoNAC72* enhanced the expression of stress-responsive genes such as *RD29A*, *RD29B*, *RD26*, *LEA14*, *ANACOR19*, *ZAT10*, *PP2CA*, and *NCED3*. These results highlight the important regulatory role of *OoNAC72* in multiple abiotic stress tolerance, and may provide an underlying reason for the spread of *O. ochrocephala*.

## Introduction

*Oxytropis ochrocephala* Bunge, one of the toxic *Oxytropis* locoweeds, distributed widely among Northwest China, where the plant often suffered from stress environment such as drought, high soil salinity and low temperature. In natural grassland plant, *O. ochrocephala* can rapidly replace local forages grass species because of its unpalatability and strong biotic stress tolerance. Grasslands infested by *O. ochrocephala* lead to tremendous losses to livestock husbandry, as well as great damage to the grassland ecological equilibrium (Zhao et al., 2013; He et al., 2015). However, existing research on *O. ochrocephala* mainly focused on surveys, allelopathy and toxicological studies of distribution (Tulsiani et al., 1988; Zhao et al., 2013), and have not yet investigated its resistance mechanism.

In general, plants often experience some harsh environments during growth and development. As a result, plants must respond to those stresses by regulating the resistance-related genes. Transcription factors play an extremely important role in the process of stresses response by activating or inhibiting the expression of the downstream target genes. In plants, several families of stress-responsive transcription factors have been functionally characterized under stress regulation networks, such as NAC, bZIP, WRKY, MYB/MYC, and AP2/ERF (Hénanff et al., 2013). NAC transcription factors, one of the largest plant-specific transcription factor families, play an important role in plant growth and stress response, and have become a hot spot in the research of gene regulation (Olsen et al., 2005b; Zheng et al., 2009). NAC transcriptional factors are derived from three kinds of genes containing particular domains of NAM (no apical meristem), ATAF (*Arabidopsis* transcription activation factor) and CUC (cup-shaped cotyledon) (Souer and Al, 1996; Aida et al., 1997).

NAC transcription factors are key regulators of plant resistance to stress by ABA-dependent or ABA-independent pathways (Puranik et al., 2012). Currently, the NAC transcription factor family have been systematically screened and analyzed in various plants, such as *Arabidopsis* (138), rice (158), wheat (134), canola, cotton, banana, and soybean (Hegedus et al., 2003; Ooka et al., 2003; Yujie et al., 2008; Meng et al., 2009; Tran et al., 2009; Tang et al., 2012; Jia et al., 2014; Jinpu et al., 2014; Tak et al., 2017). In *Arabidopsis*, Miki et al. (2010) found that *RD26/ANAC072* was significantly induced by drought, high salt and ABA, and the *rd26* mutant was not sensitive to exogenous ABA, revealing positive regulation by ABA signaling under drought stress. Similar study showed that *ANAC096* also exhibits ABA-dependent signaling and regulates the response of transgenic *Arabidopsis* to osmotic stress (Xu et al., 2013). Moreover, *ANAC096* was reported to have a synergistic relationship with ABRE binding factor and increased plant stress resistance (Xu et al., 2013). In rice, the expression of *ONAC022* was up-regulated by various stresses (Hong et al., 2016). Transgenic rice plants overexpressing *OsNAC5* and *OsNAC6* enhanced dehydration, high salinity and disease tolerances (Nakashima et al., 2007; Takasaki et al., 2010; Jeong et al., 2013). The overexpression of *OsNAC9* altered root architecture of rice plants, enhancing drought resistance and grain yield under field conditions (Redillas et al., 2012). In wheat, *TaNAC29* and *TaNAC2* can be up-regulated by different abiotic stresses, and transgenic *Arabidopsis* plants overexpressing these genes improved salt and drought tolerance (Mao et al., 2012, 2014; Huang et al., 2015; Huang and Wang, 2016). Additionally, in soybean, Tran et al., 2009 screened and cloned 31 soybean *NAC* genes, and found that nine of them were induced by drought stress. Pinheiro et al. (2009) found that the expression of *GmNAC2/3/4* was significantly induced by osmotic pressure, and Gm*NAC3/4* was simultaneously induced by ABA, JA, and salt. Although a growing number of studies have shown that NAC transcription factors play a critical regulatory role in a variety of stress-responsive signaling pathways in higher plants, the biological function of *O. ochrocephala* NAC transcription factor is still unknown.

In this study, an abiotic stress-related NAC family gene *OoNAC72* from *O. ochrocephala* were screened and characterized, and then the subcellular localization and transcriptional activation activities of OoNAC72 protein were further verified. The expression patterns of *OoNAC72* in response to polyethylene glycol (PEG), salt and exogenous ABA treatments were also determined by the quantitative real-time PCR (qRT-PCR). Moreover, transgenic *Arabidopsis* plants over-expressing *OoNAC72* (*OoNAC72*-OX) were measured for phenotypic and physiological characteristics under drought and salt stress conditions. Through this study, we aim to gain a more in-depth and comprehensive understanding of the OoNAC72 structure and its function. The results may provide a new insight into the mechanism for the rapid spread of *O. ochrocephala*.

## Materials and Methods

### Plant Materials and Growth Conditions

Mature *O. ochrocephala* seeds were collected from Haiyuan, Ningxia Province (36°29′49″N 105°36′49″E 2171 mH) in July 2014. Seeds after collection were pretreated with 98% H_2_SO_4_ for 6∼9 min, then they were washed in distilled water 4∼6 times and germinated on wet filter papers for 3 days in the dark on petri dishes. For hormone treatments, 3-week-old seedlings grown in a greenhouse were treated by spraying with 100 mM gibberellin (GA), 100 mM ethephon (ETH) and 100 mM abscisic acid (ABA) with equal volume of solution containing only 0.1% ethanol and distilled water as controls. For high salinity and drought treatments, roots of *O. ochrocephala* seedlings were soaked in 150 mM NaCl and 20% PEG-6000, respectively. *O. ochrocephala* seedlings treated with various chemicals and stress elicitors along with control plants were sampled at 0, 1, 3, 6, 12, 24, and 48 h post-treatment (hpt). All samples were frozen in liquid nitrogen and stored at −80°C for RNA extraction. Three independent biological replications were performed for each experiment.

### RNA Extraction and cDNA Synthesis

Extraction of *O. ochrocephala* total RNA was performed with the Trizol Reagent (TIANGEN, Beijing, China) according to the manufacturer’s instructions. Quality and integrity of total RNA was assessed by 1.0% agarose gel electrophoresis. RNA purity and concentration were determined on a NanoDrop^*TM*^ 2000 Spectrophotometer (NanoDrop Technologies, Wilmington, DE, United States). In order to perform RT-PCR and qRT-PCR, the first-strand cDNA was synthesized by reverse transcription using 3 μg total RNA in a 10 μl reaction volume according to the manufacturer’s instructions using the transcription kit (Thermo Fisher Scientific, Waltham, MA, United States). The cDNA was diluted 10-fold with nuclease-free water for RT-PCR and qRT-PCR.

### Cloning of *OoNAC72* and Sequence Analyses

The sequence of *OoNAC72* was obtained by our research group from the *O. ochrocephala’s* transcriptome sequencing data (He et al., 2015). Using the specific primers, we amplified the ORF of *OoNAC72* (Supplementary Table S1). The PCR condition was as follows: 3 min at 95°C; 34 cycles of 30 s at 95°C, 30 s at 55°C, and 30 s at 72°C; and then 10 min at 72°C. The resulting PCR products were cloned to the pGEM-T Easy Vector (TaKaRa, Beijing, China) and sequenced by Sangon Biotech Co., Ltd., (Shanghai, China). Multiple sequence alignment of *OoNAC72* with NAC TFs in other species was performed with DNAMAN 8.0. A phylogenetic tree was constructed using a neighbor-joining (NJ) method with 1000 bootstrap replicates in MEGA 5.0.

### Quantitative RT-PCR Analyses

Expression profiles of *OoNAC72* after different treatments were determined by qRT-PCR analyses with a pair of primers amplifying a 101-bp fragment (Supplementary Table S2). To ensure gene-specific amplification, the primers were used to amplify the *OoNAC72* gene by regular PCR and sequenced. For qRT-PCR analyses, reactions were conducted following the method of Zhuang et al. (2015a). *O. ochrocephala Histone H3* (KR733680.1) and *Actin101* (KR822225.1) were used as the internal references (He et al., 2015). *OoNAC72* expression level was calculated using the relative 2^–Δ⁢ΔCt^ method. Three replications were performed for each experiment.

### Sub-Cellular Localization of *OoNAC72*

The coding sequence without the stop codon of *OoNAC72* was transferred into the pCAMBI1302-eGFP vector (Invitrogen, United States) to generate a pCAMBI1302-OoNAC72-eGFP fusion protein using a pair of primers containing *Bgl* II or *Spe* I site (Supplementary Table S3). The re-combinational construct pCAMBI1302-OoNAC72-eGFP and pCAMBI1302-eGFP (control vector) were infiltrated into the leaves of 6-week-old *Nicotiana benthamiana*, respectively (Sheludko et al., 2007). After transformation for 36–60 h, the expression location of OoNAC72:eGFP fusion protein was observed using confocal laser scanning microscopy (CLSM, Olympus FV1000, Olympus Optical Company Ltd., Japan). Laser scanning confocal microscope was used to detect the eGFP (excitation: 488 nm, emission: 510 nm) fluorescence signal. eGFP images, The 40,6-diamidino-2-phenylindole (DAPI) (excitation: 405 nm, emission: 461 nm) nuclear stain was used to determine the location of nucleus. Images were acquired with the software FV10-ASW 4.2 Viewer. Each digital image was recorded with the same camera settings and was not further processed.

### Transcriptional Activation Analysis of OoNAC72

For transactivation analysis of OoNAC72 in yeast cell, the yeast strain AH109 (Clontech) was transformed with the appropriate bait vectors. The full-length coding sequence without the stop codon of *OoNAC72* was amplified using three pairs of primers with *Smal* I - and *Pst* I sites (Supplementary Table S3). The PCR products were digested with *Smal* I and *Pst* I and then were cloned into the GAL4 binding domain vector pGBKT7 according to the manufacture’s protocol (Clontech). Empty pGBKT7 vector was used as a negative control and the pGAL4 vector was used as a positive control. Transformed yeast cells were transformed onto SD medium (SD / -Trp, SD/ -Trp-His-Ade, SD / -Trp-His-Ade / X-α-gal) to compare their survival. Plates were incubated at 28°C for 3 days before photographing.

### Generation of Transgenic Plants

Although *O. ochrocephala* is widely distributed as a leguminous plant on the grassland, its current culture system with a complete growth cycle in the laboratory has not been reported, which makes the gene function research become extremely difficult with *O. ochrocephala* as a host plant. Therefore, *OoNAC72* was transferred into the model plant *Arabidopsis* to reveal its potential biological functions. The full-length coding sequence without the stop codon of OoNAC72 was cloned into pCAMBI1302-OoNAC72-eGFP and transformed into *Arabidopsis* thaliana Columbia-0 (WT) plants according to the floral dip method using *Agrobacterium tumefaciens* strain GV3101 (Clough and Bent, 1998). The positive transgenic lines were screened on homomycin (50 mg/L) plates, and further identified by genomic DNA PCR, and the *OoNAC72* expression level in leaves of each transgenic line was examined by qRT-PCR. The homozygous lines of T3 generation plants were used for study.

### Analysis of Stress Tolerance

Two representative transgenic lines overexpressing *OoNAC72*-OX lines and wild type (WT) *Arabidopsis* plants were selected for abiotic stress tolerance assays. For the analysis of germination rate, surface-sterilized seeds were sown on 1/2 MS solid medium supplemented with 100 and 150 mM NaCl, 200 and 250 mM mannitol, and 1 and 3 μM ABA, respectively. The seeds were first vernalized at 4°C for 3 days in dark, and then were incubated at 22°C with 16 h light / 8 h dark cycle. The germination rates of seeds were calculated when the green cotyledons emerged. For seedling root length experiment, 5-day-old seedlings cultivated on 1/2 MS solid medium were transferred onto 1/2 MS solid medium supplemented with 100 and 150 mM NaCl, 200, 250 mM mannitol or 1, 3 μM ABA for vertical culture (Yuriko et al., 2013). The length of primary roots of each subset were measured after 14 days of the treatments.

For evaluation of stresses tolerance at the vegetative growth stage, 3-week-old WT and *OoNAC72*-OX lines grown in soil under non-stress conditions were irrigated continuously with 150 mM NaCl for 30 days. For drought tolerance assay, 40-day-old plants were continuously dehydrated until the leaves withered, and then were rehydrated. Dehydration rate of detached leaves and stomatal conductance were determined on the 10th day after the onset of drought stress. To further evaluate the response mechanism of *OoNAC72*-OX lines to ABA-mediated drought, the leaves of 40-day-old seedlings of the WT and *OoNAC72*-OX lines were sheared with 10 μM ABA for 3 h (Huang and Wang, 2016; Mao et al., 2016), and then the stomatal conductance was measured under light microscopy.

### ABA Content Detection

Three-week-old *OoNAC72*-OX seedlings and WT plants grown in soil were transferred to 1/2 MS liquid medium supplemented with 20% PEG-6000 and incubated at greenhouse for 2 days. Fresh *Arabidopsis* samples harvested at different time stages during development were immediately frozen for ABA quantification by the ABA immunoassay kit (Yang et al., 2001; Yanjuan et al., 2012).

### Gene Expression Analysis of Endogenous Genes in *Arabidopsis* Leaves Under Stresses

To further investigate the molecular mechanism of stress tolerance, the expression levels of marker genes were detected in the WT and *OoNAC72*-OX plants. Total RNAs of the whole plants from 14-day-old WT and *OoNAC72*-OX plant seedlings grown on 1/2 MS solid medium supplemented with 150 mM NaCl and 200 mM mannitol were extracted with the Trizol Reagent. The qRT-PCR was performed using specific primers (Supplementary Table S4) for the expression levels of marker genes, and *Actin8* was employed as a reference control.

### Measurement of Physiological Changes

To determine water loss rate, leaves were harvested from 4-week-old seedlings of the WT and *OoNAC72*-OX plants and dehydrated on the dry filter paper (22–25°C, humidity 45–60%) for weighing at designated time points. Images were captured at 0 and 3 h after the treatment. The water loss rate was calculated based on the initial fresh weight of the leaves. Proline and malondialdehyde (MDA) content, SOD and POD activities were measured according to Wang et al. (2010) and Moore (1998). Proline content was measured according to Bates et al. (1973). At least 20 seedlings were employed for physiological indices analysis in each sample.

### Statistical Analysis

Data were presented as means ± SD of at least three independent replicates from one representative experiment. Analysis of significant difference was performed by Duncan’s multiple range tests in the ANOVA program of SPSS (IBM SPSS 22), taking ^*^P < 0.05, ^∗∗^*P* < 0.01 as critical value.

## Results

### *OoNAC72* Encodes a NAC Domain Protein

A 1301 bp cDNA containing an ORF of 996 bp (65–1060) was cloned from the transcriptome of *O. ochrocephala*. This ORF coded a protein of 331 amino acids with a theoretical molecular weight of 37.49 kDa. The conserved domain analysis revealed that its N-terminal region had a highly conserved NAC domain (amino acid 98–220), which consisted of five subdomains A–E (Supplementary Figure S1). Whereas its C-terminal region had no significant similarity to any other members of the NAC family. BLASTP analysis revealed that this protein shared the highest similarity (70%) to AtNAC72 (XP_016170502.1) in *Arabidopsis*. Further phylogenetic analysis confirmed that the relatedness of the predicted protein to AtNAC72 was highly homologous with those of *Medicago truncatula* and *Cicer arietinum* (Supplementary Figure S2). Therefore, this *O. ochrocephala* gene was designated as *OoNAC72* (MH142381).

### Sub-Cellular Localization of OoNAC72

**FIGURE 1:**
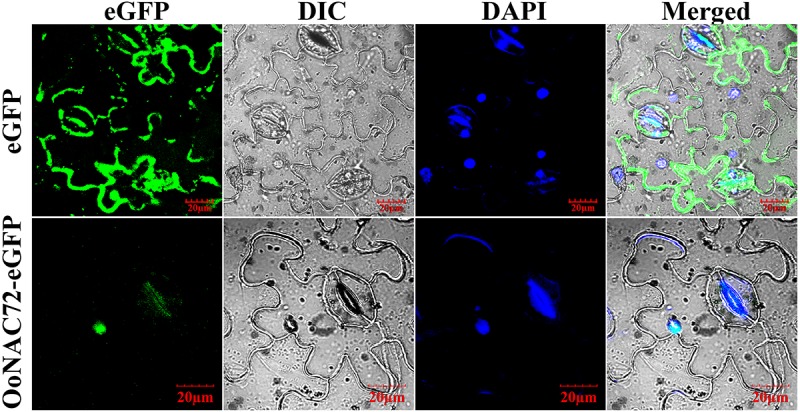
Subcellular localization of the OoNAC72 protein in leaf epidermal cells of *Nicotiana benthamiana*. eGFP and OoNAC72-eGFP constructs were separately expressed instantly in leaf epidermal cells of *Nicotiana benthamiana* and observed under a laser scanning confocal microscope after agroinfiltration for 48 h. DAPI images indicate nuclear staining. eGFP images, DAPI stained images, differential interference contrast images (DIC) and merged images were taken. Bars = 20 μm.

The sequence analysis showed that OoNAC72 possessed a conserved nuclear localization signal (NLS, positions 74–88, 117–129 aa) (Supplementary Figure S1). Meanwhile, analysis by ProtComp v.9.0 indicated a high likelihood of nuclear localization for OoNAC72 protein. To confirm this prediction, an expression cassette fusing OoNAC72 with the eGFP protein was constructed. Then the fused protein was expressed transiently in *Nicotiana benthamiana* while the pCAMBI1302-eGFP functioned as a control. Fluorescence microscopy revealed that the OoNAC72-eGFP fusion protein was exclusively localized in the nucleus in the transformed cells, whereas the control eGFP was uniformly distributed throughout the cell (Figure 1). These results further confirmed that the OoNAC72 protein was a nuclear-localized protein.

### Transcriptional Activity Assay in Yeast Cells

**FIGURE 2:**
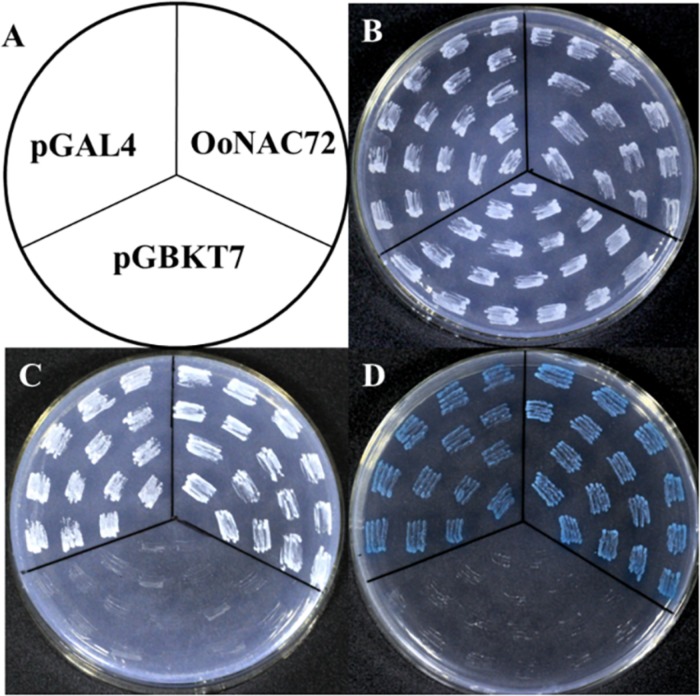
Transcriptional activation assay of the OoNAC72 in yeast cell. **(A)** Schematic Diagram. The numbers on the left indicate the last residues of polypeptides. Vectors pGBKT7 and pGAL4 were used as negative and positive control, respectively. All plasmids were transformed into the yeast strain AH109 **(B–D)**. Transformed yeasts were dripped on the SD/-Trp, SD/-Trp-His-Ade, and SD/-Trp-His-x-gal after being cultured for 3 days in the growth chamber.

The result of transcriptional activity analysis of OoNAC72 was illustrated in Figure 2. The vector pGBKT7 fused with OoNAC72 was used as the experimental set, and the empty pGBKT7 vector and pGAL4 were hired as the negative and positive controls, respectively (Figure 2A). All of the transformants grew well on selective SD/-Trp medium (Figure 2B), indicating that the three vectors were successfully transformed into the yeast cells. The GAL4-binding domain-OoNAC72 construct and pGAL4 grew well on SD/-Trp-His-Ade medium, while the transformants containing the pGBKT7 vector did not grow on the same medium (Figure 2C). The results of α-galactosidase activity assays showed that transformants containing GAL4-binding domain-OoNAC72 construct and pGAL4 appeared blue in color on SD/-Trp-His-Ade medium containing 5-bromo-4-chloro-3-indoxyl-α-D-galactopyranoside (X-α-Gal) (Figure 2D). These results indicated that OoNAC72 had transcriptional activity in yeast.

### Expression Patterns of OoNAC72 Under Various Treatments

**FIGURE 3:**
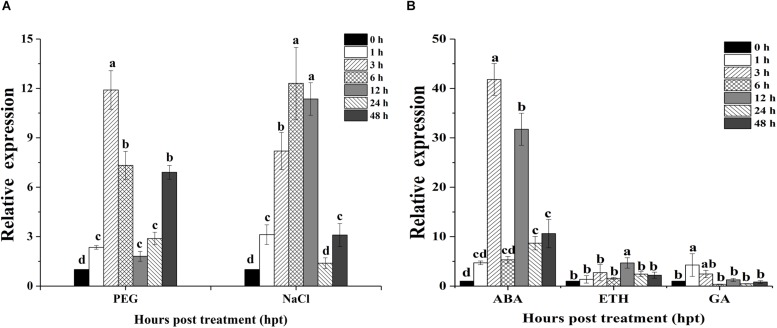
Expression patterns of *OoNAC72* in response to stress treatments. **(A)** Expression patterns under abiotic treatments: 20% PEG and NaCl. **(B)** Expression patterns under exogenous hormone treatments: ABA, ETH and GA. Three-week-old seedlings were exposed to stresses as described in section Materials and Methods. The 2^−Δ⁢Δ⁢ct^ method was used to measure the relative expression levels of the target genes in stressed and non-stressed leaves. Means are generated from three independent measurements; Error bars indicate SD, and Different letters on the histograms indicate significantly different values (^*^P < 0.05; ^∗∗^*P* < 0.01 by Duncan’s test) compared to the non-treatment controls.

Quantitative real-time PCR was used to evaluate the expression patterns of *OoNAC72* during dehydration (20% PEG-6000), salt (150 mM NaCl) and three hormone treatments (100 mM ABA, ETH, and GA) (Figure 3). When the seedlings were treated with dehydration, *OoNAC72* mRNA abundance was slightly induced at 1 h, followed by progressive elevation until reaching the peak value at 3 h, which showed an approximately 11.9-fold increase relative to the initial level (Figure 3A). Treatment with 150 mM NaCl led to a quick accumulation of *OoNAC72* mRNA level, which progressed until a maximum level was reached at 6 h, approximately 12.3-fold increase relative to the initial level (Figure 3A). Moreover, for the ABA treatment, the expression levels of *OoNAC72* increased rapidly and reached the maximum level at 3 h, being 41.8-fold greater than the control (0 h), while there were no significant changes when treated with ETH and GA (Figure 3B). All the results suggested that *OoNAC72* may respond to stress in *O. ochrocephala* by participating in ABA-dependent signal transduction pathways.

### Overexpression of *OoNAC72* Changed the Phenotype of *Arabidopsis*

**FIGURE 4:**
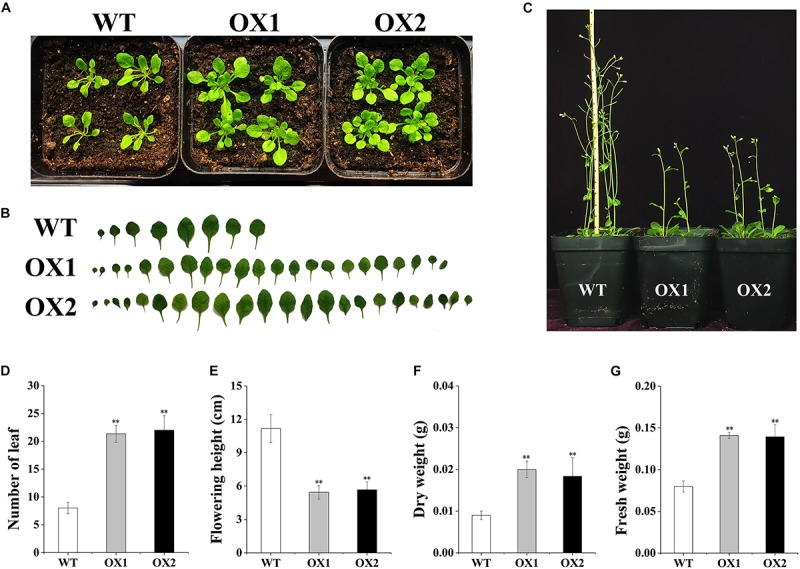
The Comparison of *OoNAC72*-OX and wild plants. **(A)** The plant phenotype of WT and *OoNAC72*-OX (OX1, OX2) plants grown under LD conditions after 3 weeks. **(B)** Schematic diagram of leaves phenotypic analyses of WT and *OoNAC72*-OX (OX1, OX2). Results from one of five biological replicates were shown. **(C)** The plant height of WT and *OoNAC72*-OX plants grown under LD conditions after 6 weeks. **(D–G)** Statistical analysis of WT and *OoNAC72*-OX (OX1, OX2) in leaf number, flowering plant height, dry and wet weight. Wet and dry weight in 3 weeks old seedlings test results. Data are represented as mean ± SD of at least three independent replications. Asterisk indicates significant difference (^*^P < 0.05; ^∗∗^*P* < 0.01) between transgenic lines and WT.

To explore the function of *OoNAC72* during the tolerance to abiotic stress, transgenic *Arabidopsis* over-expressing plants driven by the *CaMV*35S promoter were generated. We analyzed the growth status of two *OoNAC72*-OX transgenic lines (OX1, OX2) from the screening transgenic positive seedlings. Compared with wild-type plants, *OoNAC72*-OX transgenic lines (OX1, OX2) showed rosette leaves during the vegetative growth stage, and the number of leaves was significantly increased (Figures 4A,B,D); At the reproductive stage, *OoNAC72*-OX transgenic lines (OX1, OX2) showed significant delayed bolting and flowering (Figures 4C,E). *OoNAC72*-OX transgenic lines (OX1, OX2) showed no significant difference in leaf number, flowering plant height, dry and wet weight (Figures 4D–G). These findings suggested that OoNAC72 was an important regulator of plant development.

### Overexpression of *OoNAC72* Increases Tolerance to Salt and Osmotic Stresses Under Sterile Condition in *Arabidopsis*

**FIGURE 5:**
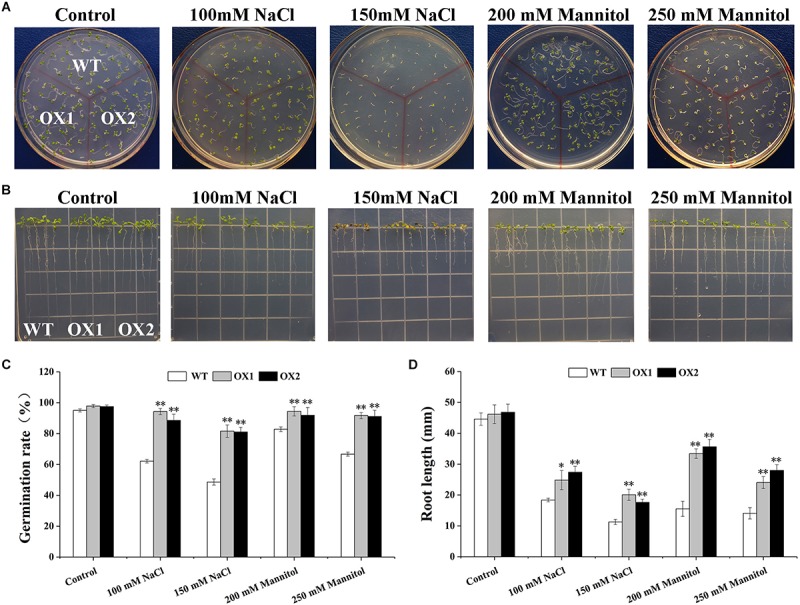
Comparison of the germination rate and root length between *OoNAC72*-OX and WT *Arabidopsis* plants. **(A)** Seed germination assay between *OoNAC72*-OX and WT *Arabidopsis* plants. The surface-sterilized seeds were sowed on 1/2 MS solid medium supplemented with 100 mM NaCl, 150 mM NaCl, 200 mM mannitol and 250 mM mannitol incubated at 22°C. **(B)** Root length assay between *OoNAC72*-OX and WT *Arabidopsis* plants. Five-day-old seedlings were cultured vertically on 1/2 MS solid medium supplemented with 100 mM NaCl, 150 mM NaCl, 200 mM mannitol and 250 mM mannitol for 7 days. **(C,D)** Effects of salt and drought stress treatments on germination rate and root length. Data are represented as mean ± SD of at least three independent replications. Asterisk indicates significant difference (^*^P < 0.05; ^∗∗^*P* < 0.01) between transgenic lines and WT.

To further investigate mechanisms of hypersensitivity to abiotic stress in *OoNAC72*-OX plants, we evaluated the stress tolerance of transgenic (OX1 and OX2 lines) and WT *Arabidopsis* in germination and root growth in seedlings (Figure 5). WT and *OoNAC72*-OX lines were no significant difference on 1/2 MS medium, whereas OX1 and OX2 lines better growth and longer root length under 150 mM NaCl, 200 mM NaCl, 200 mM mannitol and 250 mM mannitol stresses. These results suggested that *OoNAC72*-OX plants had improved tolerance to salt and drought stresses during seed germination and post-germinative growth periods.

### Drought Tolerance and Salt Tolerance Phenotypes in Transgenic Plants Under Non-sterile Conditions and Analysis of Physiological Indices

**FIGURE 6:**
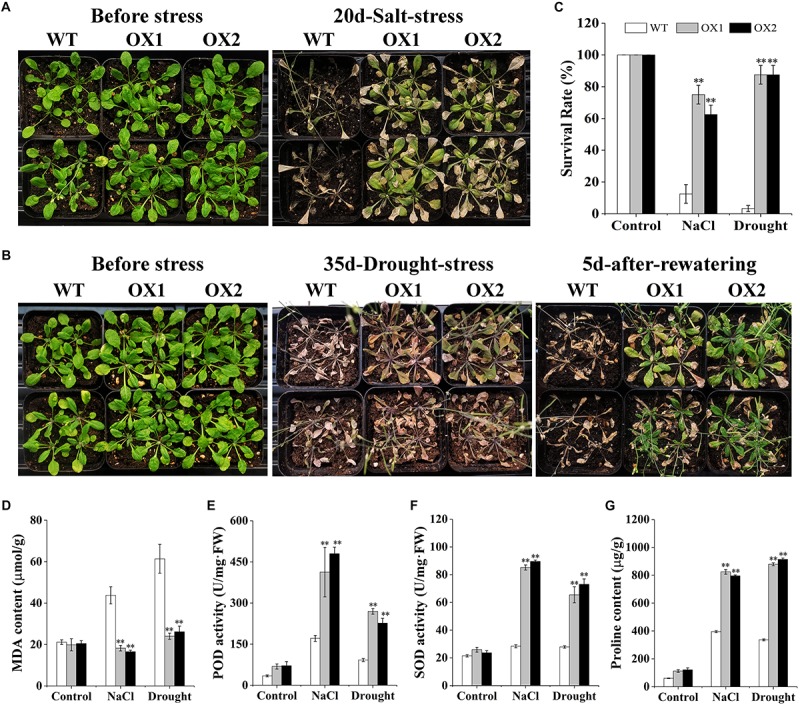
Dehydration and salt stress resistance analysis of transgenic lines. **(A,B)** Performance of *OoNAC72*-OX *Arabidopsis* plants under salt and drought stresses. Seedlings were grown without water for 35 days and re-watered for 5 days. **(C)** Survival rate after recovery from drought and salt stress. Each experiment comprises 20 plants. **(D–G)** Proline and MDA content, SOD and POD activities in WT and *OoNAC72-*OX lines after drought and salt stress. Each data point is the mean of three biological replicates. Error bars indicate SD, and asterisks indicate a significant difference (^*^P < 0.05; ^∗∗^*P* < 0.01) compared with WT.

To further elucidate the possible involvement of *OoNAC72* in the response to drought and salt, we compared drought and salt tolerance of the *OoNAC72-*OX lines and WT plants at the vegetative growth stage (Figure 6). After 20 days of 150 mM NaCl stress treatment, the phenotypes of the WT and OX1 and OX2 lines began to show significant differences, in which 87.5% of the WT died, while the majority of OX1 and OX2 lines remained green,whereas OX1 and OX2 showed significantly higher survival rate 75 and 62.5% (Figures 6A,C). In order to detect the drought resistance of *OoNAC72-*OX lines, WT and *OoNAC72-*OX lines were simultaneously withholding water for 35 and 5 days after rehydration, the phenotypes of the WT and *OoNAC72*-OX lines began to show significant differences, the leaves of WT lines completely wilted and the plants survival rate was 3.33%. However, OX1 and OX2 lines survival rate were 87.5% (Figures 6B,C). Furthermore, there were no significant differences in proline and MDA content, POD and SOD activities between WT and OX1 and OX2 lines under normal condition (Figures 6D–G). Whereas, under salt and drought stresses, OX1 and OX2 lines exhibitedhigher levels of proline content, POD and SOD activities, and lower MDA level compared with those of WT (Figures 6D–G). Phenotypic analysis showed that the *OoNAC72-*OX plants had the highest drought and salt tolerance. These data demonstrated that the *OoNAC72*-OX plants exhibited increased tolerance to salt and drought stresses, thus we speculated *OoNAC72* plays a critical role in *O. ochrocephala* response to salt and drought stresses.

### Drought Resistance of WT and *OoNAC72*-OX Plants

**FIGURE 7:**
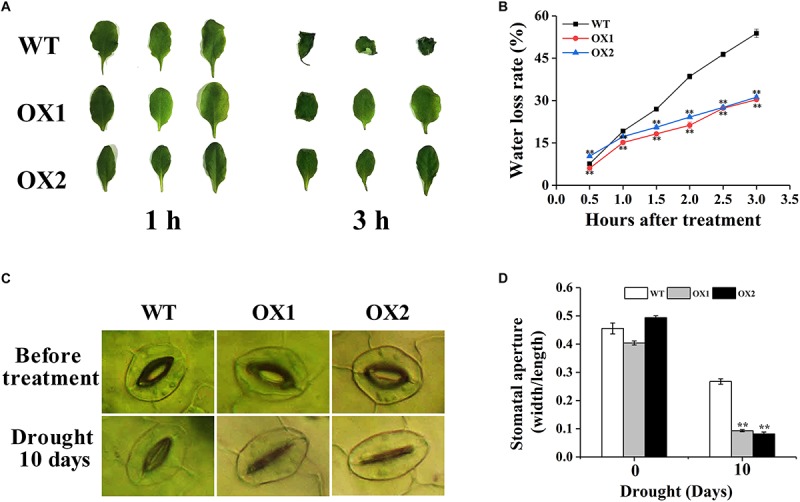
*OoNAC72*-OX plants exhibit reduced tolerance to water deficiency. **(A)** Phenotypes of detached leaves under water deficiency treatment for 1 and 3 h. **(B)** Water loss from detached rosettes of WT and *OoNAC72*-OX plants. Values are means ± SD of three independent experiments. **(C)** Stomatal apertures of WT and *OoNAC72*-OX plants. Scale bars = 30 μm. **(D)** Stomatal aperture width/length ratios under dehydration conditions in 10 days. The stomatal aperture was measured by microscope. Values are means ± SD (*n* = 50) in assays. Asterisks indicate statistically significant differences from WT. ^*^P < 0.05, ^∗∗^*P* < 0.01.

To further investigate drought sensitivity of the *OoNAC72*-OX plants, the rate of nature water loss and stomatal apertures of leaves from the 25-day-old soil-grown WT and *OoNAC72*-OX plants were detected (Figure 7). After 3 h of air drying, the leaves of OX1 and OX2 were slightly curled, while the WT plants were severely curled (Figure 7A). Additionally, *OoNAC72*-OX lines showed lower water loss rate compared with WT plants (Figure 7B), indicating that over expression of *OoNAC72* had increased water retention capacity in *Arabidopsis*. After 10 days of drought control, stomatal apertures index of the transgenic and WT lines all appeared significant changes. OX1 and OX2 plants decreased from 0.40 and 0.49 to 0.09 and 0.08, which was significantly smaller than WT (from 0.46 to 0.27) (Figures 7C,D). WT and *OoNAC72*-OX plants were treated with 20% PEG-6000 simulated drought stress. ABA was detected at different time points. The ABA content of these *OoNAC72*-OX lines were significantly higher than that of the WT (Supplementary Figure S3). Those results indicated that *OoNAC72-*OX plants may reduce the loss of water by regulating the stomata closure to improve drought resistance.

### Altered Expression of Stress-Responsive Genes in Transgenic *OoNAC72* Plants

**FIGURE 8:**
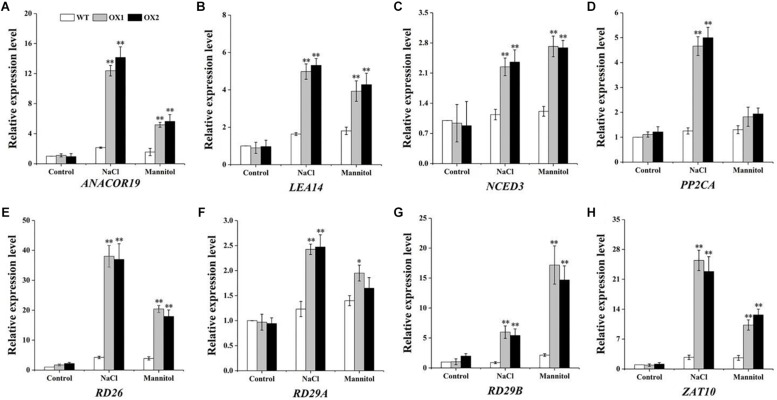
Expression levels of the stress-associated genes in WT and *OoNAC72*-OX plants. The 14-day-old seedlings grown on 1/2 MS medium were subsequently submerged in 1/2 MS cultures supplemented with 150 mM NaCl and 200 mM mannitol for 7 days. **(A–H)** Transcript levels of stress-associated genes were determined against with *ACTIN8*. Data are means ± SD of three biological replicates. Asterisks indicates insignificant differences from WT (^*^P < 0.05; ^∗∗^*P* < 0.01).

To explore the underlying basis of this phenotype caused by drought or salt stress in the transgenic plants, eight stress-responsive genes: *RD29A* (Nakashima et al., 2006), *RD29B* (Nakashima et al., 2006), *RD26* (Fujita et al., 2004), *LEA14* (Jia et al., 2014), *ANACOR19* (Tran et al., 2004), *ZAT10* (Mittler et al., 2002), *PP2CA* (Yoshida and Hirayama, 2006) and *NCED3* (Iuchi, 2001) were selected for expression pattern assays (Figure 8). Under normal condition, the expression levels of eight genes showed no significant difference between the *OoNAC72*-OX lines and WT. However, the expression levels of the eight genes in the OX1 and OX2 lines were significantly higher than those in WT plants under salt and drought conditions.

### Increased ABA Sensitivity in *OoNAC72*-OX Plants

**FIGURE 9:**
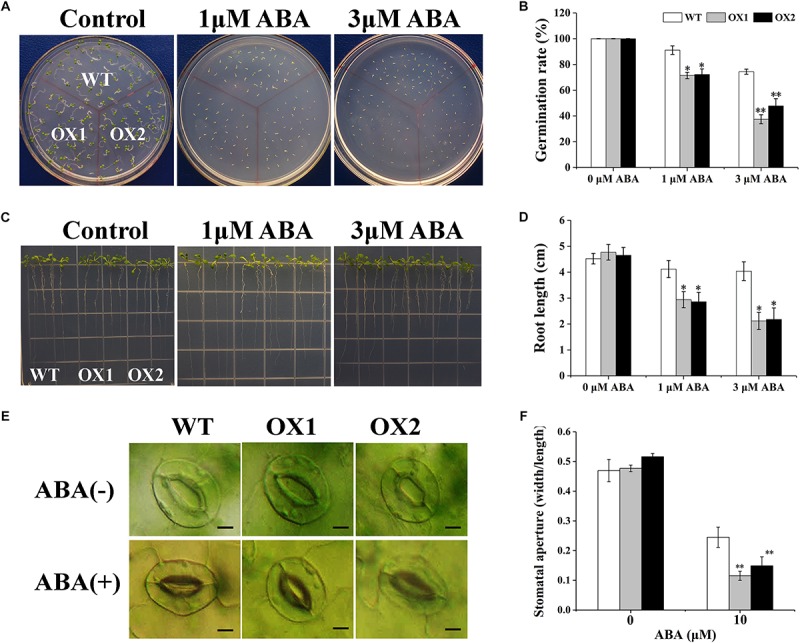
Responses of *OoNAC72*-OX plants to ABA. **(A,B)** Seed germination assay between *OoNAC72*-OX and WT *Arabidopsis* plants. The surface-sterilized seeds were sowed on 1/2 MS solid medium supplemented with 1 and 3 μM ABA incubated at 22°C. **(C,D)** Root length assay between OoNAC72-OX and WT *Arabidopsis* plants. Five-day-old seedlings were cultured vertically on 1/2 MS solid medium supplemented with 1 and 3 μM ABA for 7 days. **(E,F)** Stomatal closure of WT and *OoNAC72*-OX plants with 10 μM of ABA. Values are means ± SD (*n* = 50) in assays. Asterisks indicate statistically significant differences from WT. ^*^P < 0.05, ^∗∗^*P* < 0.01.

The ABA sensitivity of the *OoNAC72*-OX lines was assessed by analyses of seed germination and seedling growth (Figure 9). In the absence of ABA, no obvious difference was observed between transgenic and WT plants under normal growing conditions. However, when supplied with 1 or 3 μM ABA, seeds germination and seedling root length of the OX1 and OX2 lines were significantly inhibited compared with the WT lines (Figures 9A–D). Moreover, when 40-day-old plants were treated with 10 μM ABA, the stomatal apertures of both wild-type and *OoNAC72*-OX lines all happened to change (Figure 9E). The stomatal apertures index of WT plants decreased from 0.47 to 0.25, while the OX1 and OX2 lines dropped from 0.48 and 0.52 to 0.12 and 0.15 (Figure 9F). It indicated that the *OoNAC72*-OX lines were more rapid and variable in stomatal conductance. These results indicated that overexpression of *OoNAC72* gene led to increased ABA sensitivity, which resulted in retarded growth of transgenic plants.

## Discussion

### *O. ochrocephala* Can Be Exploited as a Pasture and Ecological Resource

*O. ochrocephala* is a perennial poisonous plant widely distributed in harsh environments, and livestock generally do not eat it when edible pasture is relatively abundant. In recent years, due to the emergence of extreme climatic and human overgrazing, *O. ochrocephala* has spread rapidly and become one of the main poisonous weeds in Western China. Studies have shown that *O. ochrocephala* is rich in protein and mineral elements and can be used as a good forage forb for livestock after detoxification (Shen and Mo, 2017). At the same time, as a highly resistant plant, *O. ochrocephala* has important ecological value in wind break and sand fixation, as well as biodiversity maintenance. In view of these characteristics, we focus on *O. ochrocephala* to elucidate the survival mechanism in adversity, and ultimately to provide a theoretical basis for its development and utilization.

### *OoNAC72* Is a Stress-Related NAC Transcription Factor

In the present study, we characterized the *OoNAC72* gene, a novel stress-related member of the NAC gene family in *O. ochrocephala*. OoNAC72 protein contains a typical NAC conserved domain located in the N-terminal region, which can be divided into five subdomains. As the transcriptional regulatory domain, the C-terminus is extremely variable. This result is in agreement with previous reports on other NAC TFs domains, such as *PbeNAC1*, *ZmNAC55*, *CmNAC1* (Mao et al., 2016; Cao et al., 2017; Jin et al., 2017). Phylogenetic analysis confirmed the *OoNAC72* had the highest homology with the two legumes, *Medicago truncatula* and *Cicer arietinum*. Subcellular localization and transactivation analysis jointly revealed that OoNAC72 was a transcription factor, which was consistent with previous research (Mao et al., 2016). Therefore, OoNAC72 act as a transcriptional activator, depending on interactions with other transcription factors or conformational changes. These results speculate that *OoNAC72* is a novel stress-related member of the NAC gene family in *O. ochrocephala*.

### *OoNAC72* Is Involved in Response to Diverse Stresses

In previous researches, many NAC genes had been identified as an important part of the progress of complex signal transduction when plants were subjected to various stresses, especially abiotic stress such as drought and salt stress (Puranik et al., 2012). In our study, we found that *OoNAC72* can be induced by high salt, drought and ABA treatment, whereas not induced by GA and ETH. we speculated that *OoNAC72* may respond to stress in *O. ochrocephala* by ABA-dependent signal transduction pathways. These abiotic stresses induced expression pattern of *OoNAC72* was supported by earlier studies, such as in pumpkin (Cao et al., 2017), *Pyrus betulifolia* (Jin et al., 2017), tomato (Zhu et al., 2014), chickpea (Yu et al., 2014, 2016) and *Tamarix hispida* (Wang et al., 2017).

### *OoNAC72* Is Involved in Developmental Processes

Numerous reports have demonstrated that NAC TFs are involved in a number of biological processes, such as regulating the growth of plant cells (Hiroaki et al., 2010), seed development (Sperotto et al., 2009), embryonic development, fiber formation and development, cell differentiation and leaf senescence (Youn-Sung et al., 2007). In our study, we found that *OoNAC72*-OX (OX1, OX2, OX3) lines showed significant rosette leaves, delayed twitches and flowering compared with WT plants. However, the mechanism resulting from overexpression of *OoNAC72* in regulating this phenotype is still unknown and waiting for further research.

### Overexpression of *OoNAC72* Improves Stress Tolerance in *Arabidopsis*

Overexpression or deletion mutation is the primary way to study the function of NAC transcription factors in adversity stress. In this study, several physiological changes of *OoNAC72*-OX lines seem to be involved in abiotic stress-resistant and molecular mechanisms. Firstly, in terms of phenotype, *OoNAC72*-OX lines appeared improved tolerance to salt and drought stresses during seed germination, post-germinative growth periods and vegetative growth stage. Consistent with the results of *OoNAC72*, *CarNAC4*-transgenic plants exhibited enhanced drought and salt tolerance than the WT plant, which were strongly demonstrated by both morphological and physiological changes (Yu et al., 2016). Secondly, at the physiological and biochemical levels, proline and MDA content, POD and SOD activities in plants overexpressing *OoNAC72* have been significantly induced under different stress conditions. Thirdly, at the molecular level, our data also demonstrated that over-expression of *OoNAC72* enhanced the expression of stress-responsive genes such as *RD29A*, *RD29B*, *RD26*, *LEA14*, *ANACOR19*, *ZAT10*, *PP2CA*, and *NCED8*. Previous studies have confirmed that NAC proteins could bind to NACRS containing the core sequence “CGT[A/G]” to regulate gene expression through ABA dependent pathway for various stress response (Duval et al., 2002; Olsen et al., 2005a; Takasaki et al., 2010; Jensen et al., 2013). However, the genome of *O. ochrocephala* has not been sequenced and the *OoNAC72* promoter sequence could not be cloned, and so it is impossible to predict these *cis*-acting elements associated with stress in the promoter, and resistance mechanisms need to be further investigated.

### *OoNAC72* Functions in ABA Signaling and Confers Drought Resistance to Transgenic Plants

Abscisic acid is a very important signaling molecule that respond to many adverse environmental stresses such as high salt, drought and extreme temperatures (Agarwal and Jha, 2010; Fujita et al., 2011). In plants, higher ABA sensitivity may irritate the stomata to maintain moisture and enhance drought resistance. In our study, ABA content of WT and *OoNAC72*-OX plants treated with 20% PEG-6000 simulating drought stress showed that ABA content of the *OoNAC72*-OX lines was significantly higher than that of the WT (Supplementary Figure S3). Furthermore, we found that the stomata in *Arabidopsis* overexpressing *OoNAC72* had different degrees of closure after drought stress, indirectly mapping the relationship between drought stress and the ABA pathway. Similar results were found in *Arabidopsis* and maize (Ping-Li et al., 2007; Mao et al., 2016). Overexpression of *KUP6* (K+ uptake transporter 6) in *Arabidopsis* increased the sensitivity of ABA to drought stress through faster stomatal closure, thereby improving the tolerance of transgenic plants to drought stress (Yuriko et al., 2013). Genes such as *PP2Cs*, *SnRK2s*, *NAC* and *WRKY* played a vital role in ABA signaling pathways induced by drought stress (Kim, 2014). Therefore, we supposed that *OoNAC72* may enhance the drought resistance of transgenic *Arabidopsis* by participating in the ABA pathway.

### *OoNAC72* Scavenges ROS Capability by Increasing ABA Content Under Stress

Under stress conditions, plants reduce the damage caused by stress-induced ROS by enhancing their antioxidant defense system (Oracz et al., 2009; Suzuki et al., 2012). MDA is one of the important parameters to measure the degree of oxidative damage in plant cells. While pro contributes to osmotic adjustment to effectively enhance the antioxidant system and reduce peroxidative damage (Zhuang et al., 2015a). In our study, transgenic plants produced less MDA and more pro under stress than wild-type plants, revealing that *OoNAC72* overexpression enhanced the body’s regulation of feedback regulatory substances.

Under drought stress, ABA acts as an upstream signaling of NO and they form cross-signal pathways co-regulate a balance between ROS (H_2_O_2_) and NO production (Zhuang et al., 2015b). Our resistance assays showed that the ABA contents, and SOD and POD activities were significantly induced by *OoNAC72* under drought stresses condition. The increase in ABA content leads to an increase in the concentration of NO for activating NO signaling pathway. On the one hand, the low concentration of NO interacts with H_2_O_2_ to stimulate the antioxidant defense system. On the other hand, the increase of SOD and POD activity further enhances plant’s ability to scavenge excessive H_2_O_2_. The synergy between NO signal and ROS signal promotes the dynamic balance between NO and H_2_O_2_, and ultimately improves plant resistance tolerance (Miller et al., 2010). These results indicate that showing the positive regulation of *OoNAC72* on ABA signaling pathway under drought stress.

## Conclusion

Plant NAC transcription factors control diverse biological processes, such as differentiation, development and abiotic stress responses. In this study, we identified a gene encoding NAC72-type transcription factor from *Oxytropis ochrocephala*, and characterized its role. The nuclear localization and transcriptional activity of this protein indicate the possible function of *OoNAC72* as a transcription factor. In addition, expression of *OoNAC72* transcript was shown to be up-regulated in response to abiotic stresses and exogenous ABA. Furthermore, analyses of transgenic *Arabidopsis* expressing *OoNAC72* also supported the involvement of OoNAC72 in drought and salinity responses as well as in the regulation of ABA-dependent processes.

## Author Contributions

HG and YF conceived and designed the study. HG, XL, and FN performed the experiments. HG, XL, and QZ analyzed the data and wrote the manuscript. FN, QZ, NF, DC, and DM reviewed the manuscript. WH, BG, YW, and YF contributed reagents, materials, and fund support.

## Conflict of Interest Statement

The authors declare that the research was conducted in the absence of any commercial or financial relationships that could be construed as a potential conflict of interest.
