# Supplementary material for: OoNAC72, a NAC-Type Oxytropis ochrocephala Transcription Factor, Conferring Enhanced Drought and Salt Stress Tolerance in Arabidopsis
Source: Front Plant Sci. 2019 Jul 11;10:890. doi: 10.3389/fpls.2019.00890 (PMC6637385; doi:10.3389/fpls.2019.00890)
Supplement: Supplementary file 1 [file Data_Sheet_1.docx]

Supplementary Tables and Figures

OoNAC72, a NAC-type *Oxytropis ochrocephala* Transcription Factor， Conferring Enhanced Drought and Salt Stress Tolerance in *Arabidopsis*

**Huirui Guan**^†^**, Xin Liu**^†^**, Fei Niu, Qianqian Zhao, Na Fan, Duo Cao, Dian Meng, Wei He, Bin Guo, Yahui Wei^*^, Yanping Fu^*^**

Department of Life Science, Key Laboratory of Resource Biology and Biotechnology in Western China, Northwest University, Xi’an, China

*** Correspondence:** Yahui Wei: [weiyahui@nwu.edu.cn](mailto:weiyahui@nwu.edu.cn);

Yanping Fu: [fuyanping@nwu.edu.cn](mailto:fuyanping@nwu.edu.cn);

# Supplementary Tables

**Supplementary Table 1 | Primers used for cloning cDNA of *OoNAC72* from *O. ochrocephala***

| **Gene Name** | **Forward/Reverse primers (5'-3')** | **Tm** |
| --- | --- | --- |
| *OoNAC72* | F: CACAGTGATTTAGAATGGGAGTT | 56℃ |
|  | R: ATTAAATGGTGGCATCATTAACT |  |

**Supplementary Table 2 | Primers used for qRT-PCR analysis of *OoNAC72* gene expression**

| **Gene Name** | **Forward/reverse primers (5'-3')** | **Tm** |
| --- | --- | --- |
| *OoNAC72* | F: CAACTAGCTCCACTCCACCTAC | 56℃ |
|  | R: AACTCCCATTCTAAATCACTGTG |  |
| *Histone H3* | F: ATGGGCTTGGTGCTGTTA | 55℃ |
|  | R: CCTTCATCAGACTAGCAGTAAAC |  |
| *Actin101* | F: TTATGTCCTTGAAAGCCAGA | 55℃ |
|  | R: GAGACTTATCCTCCCATCCT |  |

**Supplementary Table 3 | Primers used in subcellular localization assay and transactivation activity analysis of OoNAC72**

| **Gene Name** | **Forward/reverse primers (5'-3')** | **Restriction enzymes** |
| --- | --- | --- |
| *OoNAC72* | GAagatctATGGGAGCTCCAGAGAGAGAC | *Bgl* II |
| *OoNAC72* | GactagtTTGCCCGAACCCAAACC | *Spe* I |
| *OoNAC72-BD* | TcccccgggTATGGGAGCTCCAGAGAGAGAC | *Smal I* |
| *OoNAC72-BD* | AActgcagGTCATTGCCCGAACCCAA | *Pst I* |

**Supplementary Table 4 | Primers used in qRT-PCR for gene expression analyses of marker genes**

| **Gene Name** | **Gene ID** | **Forward/reverse primers (5'-3')** |
| --- | --- | --- |
| *Actin8* | At1g49240 | CTCAGGTATTGCAGACCGTATGAG  CTGGACCTGCTTCATCATACTCTG |
| *RD26* | AT4G27410 | GAAGGTGAGGCGGAGAGTG  CCCGAAACTCTGAGTCAACCT |
| *COR47* | NM101894 | TCCCAGGACACCACGACAAGAC  CCTCTTCAGTGGTCTTGGCATG |
| *RD29A* | AT4G27410 | TGGACACGAATTCTCCATCA  TTCCAGCTCAGCTCCTGATT |
| *LEA14* | AT1G01470 | GATTTCTTCTGATCGACAAAACCTA  AGCAAACCCAACTTATTACATTACG |
| *ZAT10* | AT1G27730 | GGAGTCAGCGAGGTGGAAGT  TGCTCCTTCGGTCTGTCCTAA |
| *RD29B* | AT5G52300 | GGAGAGAGCAGAGAGGCTCA  CCGTTGACCACCGAGATAGT |
| *ANAC019* | AT1G52890 | CGCTAACTGCGGTGACTCTA  CAATCCTCGCAGCTTCATCT |
| *NCED3* | AT3G14440 | TCAAAACCGGTGAATCAACT  TTCGTCTTCCTCTCCTCCTTC |
| *PP2CA* | AT3G11410 | ATGTCAGAGACCAACAAGAATGCC  CTACTTGTTCAGGCCGGTCTTG |

# Supplementary Figures


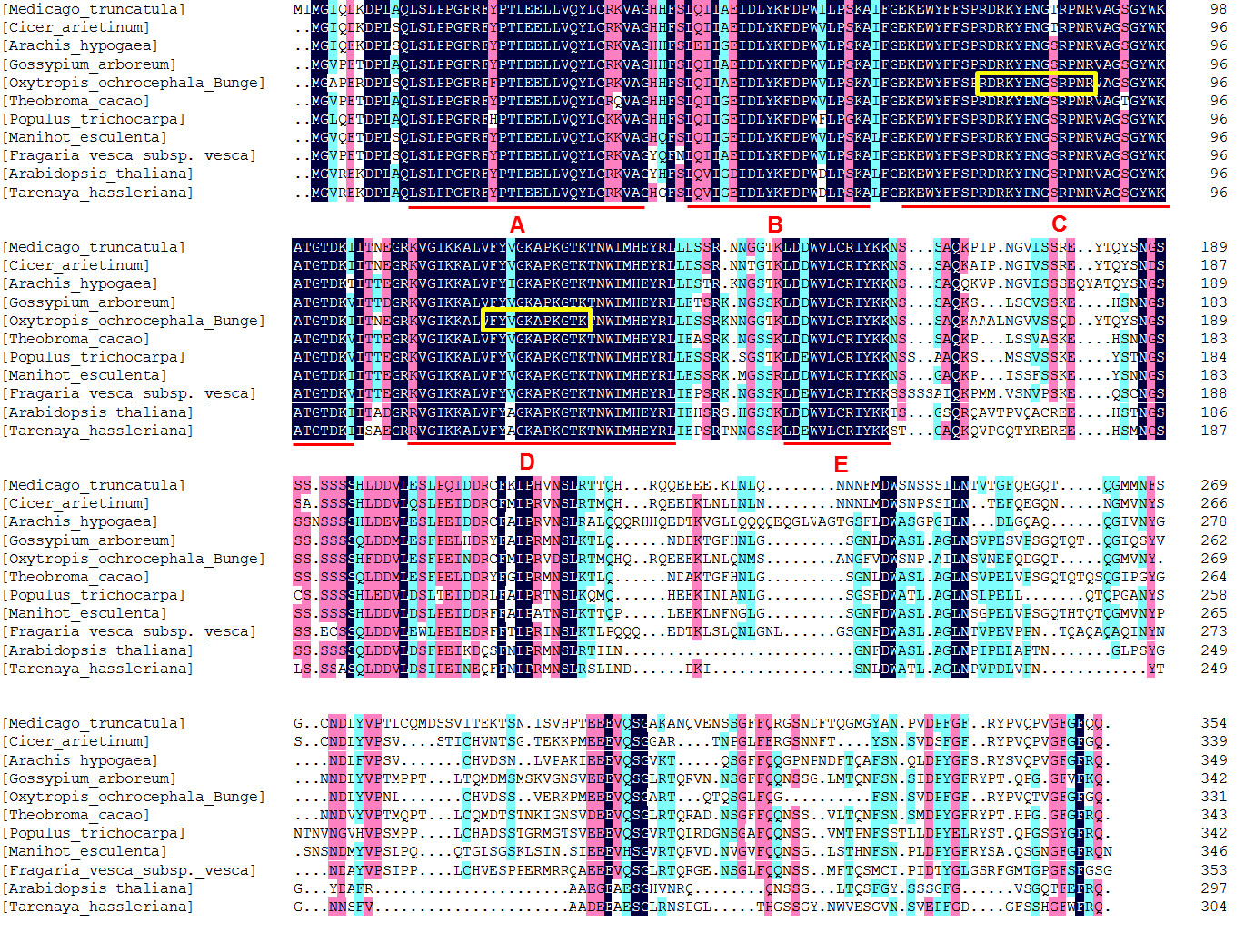


**Supplementary Figure S1 | Multiple alignment of the amino acid sequences of OoNAC72 with NAC proteins from different species.** A B C D and E represent the five subdomains of NAC domain; C and D subdomains framed in the yellow box indicates the nuclear localization signal.


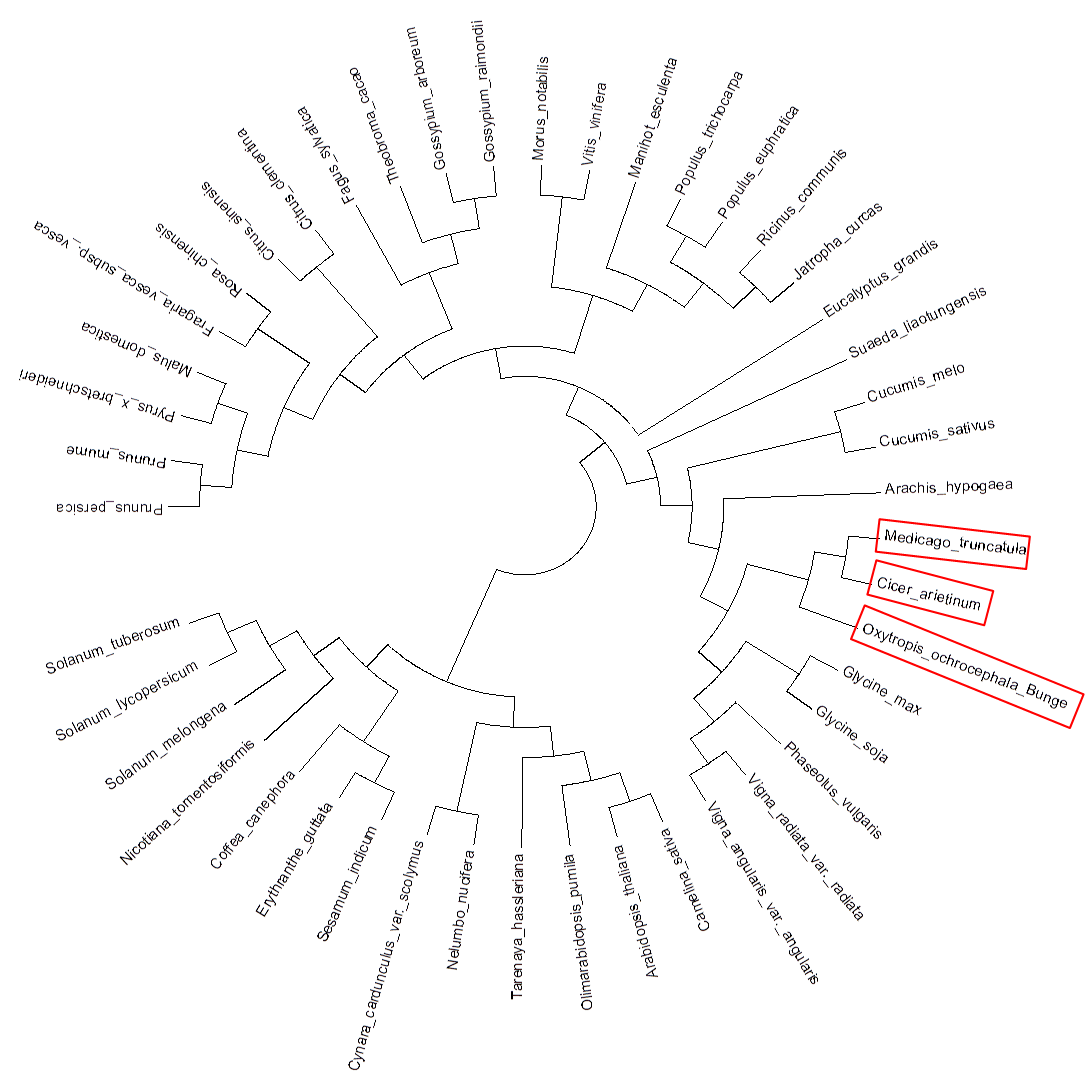


**Supplementary Figure S2 | Phylogenetic analysis of the deduced amino acid sequences of OoNAC72 with other NAC domain proteins.**


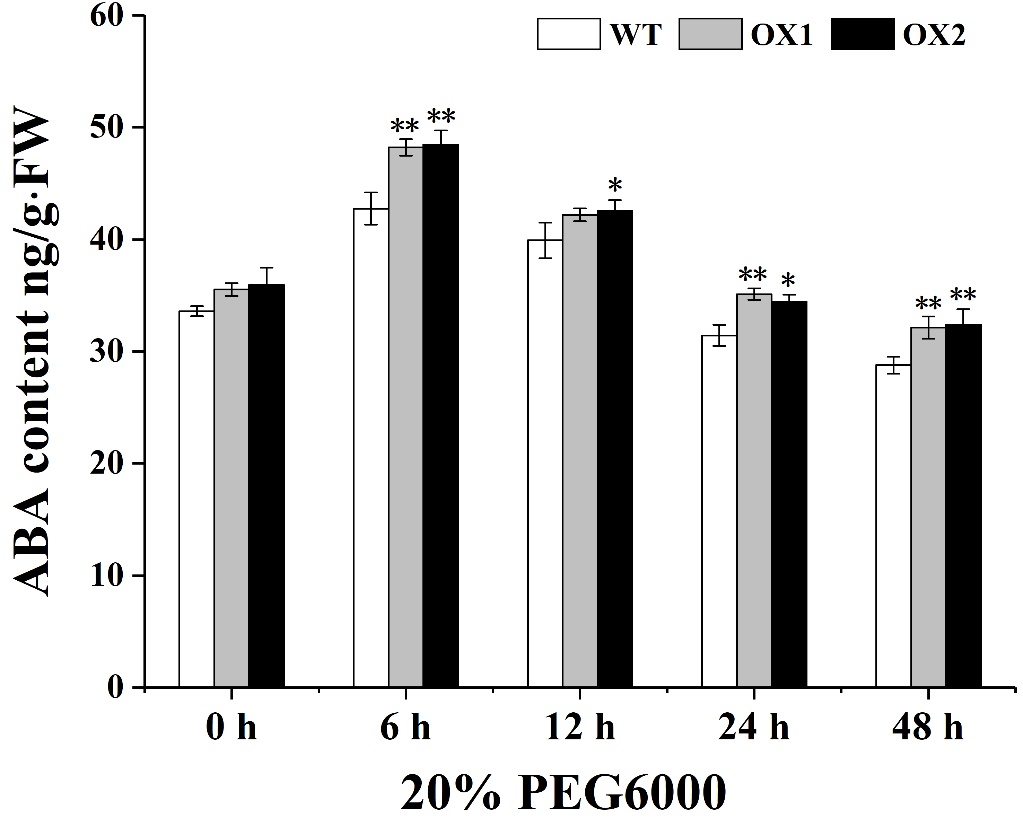


**Supplementary Figure S3 | ABA content as determined by ELISA. Values are the mean ± SD (n = 3 experiments, * P < 0.05). FW, fresh weight.**
